# The radiosensitizer Onalespib increases complete remission in ^177^Lu-DOTATATE-treated mice bearing neuroendocrine tumor xenografts

**DOI:** 10.1007/s00259-019-04673-1

**Published:** 2020-01-07

**Authors:** Sara Lundsten, Diana Spiegelberg, Nakul R. Raval, Marika Nestor

**Affiliations:** 1 Department of Immunology, Genetics and Pathology, Uppsala University, The Rudbeck Laboratory, SE-751 85 Uppsala, Sweden; 2 Department of Surgical Sciences, Uppsala University, Uppsala University Hospital, Entrance 70, SE-751 85 Uppsala, Sweden

**Keywords:** Neuroendocrine cancer, ^177^Lu-DOTATATE, HSP90, Onalespib, Radiosensitization

## Abstract

**Purpose:**

^177^Lu-DOTATATE targeting the somatostatin receptor (SSTR) is utilized for treatment of neuroendocrine tumors (NETs). Onalespib, a heat shock protein 90 (HSP90) inhibitor, has demonstrated radiosensitizing properties and may thus enhance the effect of ^177^Lu-DOTATATE. Consequently, the aim of this study was to assess the potential of Onalespib in combination with ^177^Lu-DOTATATE in vivo and to examine the toxicity profiles of the treatments.

**Methods:**

^177^Lu-DOTATATE selectivity and distribution in NET xenografts were studied using biodistribution and autoradiography. Therapeutic effects of Onalespib in combination with ^177^Lu-DOTATATE were studied in NET xenografts. Histological analyses were used to assess molecular effects from treatment and to establish toxicity profiles.

**Results:**

Biodistribution and autoradiography confirmed the SSTR-selective tumor uptake of ^177^Lu-DOTATATE, which was unaffected by Onalespib treatment. Immunohistochemistry verified molecular responses to Onalespib therapy in the tumors. While Onalespib and ^177^Lu-DOTATATE monotherapies resulted in a 10% and 33% delay in tumor doubling time compared with control, the combination treatment resulted in a 73% delayed tumor doubling time. Moreover, combination treatment increased complete remissions threefold from ^177^Lu-DOTATATE monotherapy, resulting in 29% complete remissions. In addition, histological analyses demonstrated radiation-induced glomerular injury in the ^177^Lu-DOTATATE monotherapy group. The damage was decreased tenfold in the combination group, potentially due to Onalespib-induced HSP70 upregulation in the kidneys.

**Conclusion:**

Treatment with Onalespib potentiated ^177^Lu-DOTATATE therapy of NET xenografts with a favorable toxicity profile. Utilizing Onalespib’s radiosensitizing properties with ^177^Lu-DOTATATE may lead to better therapeutic results in the future and may reduce unwanted side effects in dose-limiting organs.

**Electronic supplementary material:**

The online version of this article (10.1007/s00259-019-04673-1) contains supplementary material, which is available to authorized users.

## Introduction

Tumors that arise from the neuroendocrine system can occur throughout the body, but the most common sites of the primary disease are the gastrointestinal (GI) tract and the lungs [1]. Neuroendocrine tumors (NETs) consist of around 2% of all cancer cases and there is a wide range in malignancy within this disease group [1, 2]. While early-stage disease is regularly surgically resected, patients diagnosed at a later stage frequently present with metastases. For this patient group, curative surgery is rarely an option [1, 2].

Instead, peptide receptor radionuclide therapy (PRRT) targeting the somatostatin receptors (SSTRs) has revolutionized the treatment of metastatic or inoperable NETs [3]. SSTRs are overexpressed in a variety of cancers where the highest abundance of around 80% is found in well-differentiated NETs originating from the GI tract and pancreas [4]. The SSTRs can be targeted with somatostatin analogues conjugated to a therapeutic radionuclide [5]. The systemically administered radiopeptide can deliver local radiotherapy to both primary tumors as well as metastases. The SSTR-targeting radiopeptide ^177^Lu-DOTA-(Tyr^3^)-Octreotate (^177^Lu-DOTATATE or Lutathera®), with highest affinity to SSTR2 [6], was approved for therapy of gastroenteropancreatic NETs by the European Medicines Agency and the Food and Drug Administration after the NETTER-1 trial [3, 7, 8]. Clinical studies investigating the efficacy of ^177^Lu-DOTATATE have demonstrated improved response rates, however rarely resulting in complete response [3, 9–12]. This may be due to the fact that the doses required to completely eradicate the lesions are not manageable from a toxicity perspective.

The development of PRRT targeting SSTRs has involved studies with various radionuclides, i.e., ^90^Y and ^177^Lu [13]. It was concluded that the latter provided a more manageable renal toxicity profile due to shorter penetration depth [13]. Moreover, co-infusion of amino acids has been included in the renal protection regimen as it inhibits tubular reabsorption of the radiopeptide [13]. Although kidney toxicity is considered manageable today, the kidneys, along with bone marrow, are still the main dose-limiting organs [13]. Consequently, there is a need for further development of the treatment for patients with inoperable NETs, in order to further improve curative rates without causing unnecessary toxicity.

The concept of radiosensitization is an emerging field within cancer medicine. The addition of radiosensitizing drugs can influence the tumor cells’ ability to respond to radiation and further increase the efficacy of the therapy, e.g., by influencing DNA damage and repair mechanisms [14]. One proposed approach to potentiate the therapeutic response of radiotherapy is by inhibition of heat shock protein 90 (HSP90) [15, 16]. HSP90 is a molecular chaperone that is overexpressed in tumor cells, reaching levels up to 10-fold to those of normal tissue, where it helps fold, refold, and protect its client proteins from degradation. The list of HSP90 client proteins is long and includes proteins involved in proliferation, DNA repair and angiogenesis, such as EGFR and VEGFR [17, 18]. In addition, HSP90 inhibition may also lead to upregulation of a related heat shock protein, HSP70 (HSP72, HSPA1A) [19–21], a protein thought to play a protective role in renal damage [22–24].

In neuroendocrine cancers, HSP90 has been reported to be a potential therapeutic target, due to high expression in both primary tumors and metastases [25, 26]. A number of HSP90 inhibitors, including the second-generation small-molecule inhibitor Onalespib (AT13387), have demonstrated antitumorigenic effects on neuroendocrine tumor cells in vitro [27, 28]. Preclinical studies by our group have demonstrated that Onalespib can act as a radiosensitizer when combined with external beam radiotherapy (EBRT) in vitro as well as in vivo [15, 29], where HSP90 inhibition with Onalespib in combination with EBRT resulted in downregulation of DNA repair proteins, e.g., DNA-PKcs and ATM [15]. Moreover, we have recently demonstrated the potential of combining ^177^Lu-DOTATATE with Onalespib in vitro [30], and another HSP90 inhibitor was recently shown to reduce tumor growth when combined with external radiotherapy or ^177^Lu-DOTATATE in small intestine NET xenografts [16]. While previous studies support the feasibility of utilizing HSP90 inhibitors such as Onalespib to potentiate effects of ^177^Lu-DOTATATE, none has investigated the molecular, therapeutic, and toxicity effects of such a combination in vivo.

The aim of this study was to evaluate HSP90 inhibitor Onalespib as a therapeutic strategy to potentiate ^177^Lu-DOTATATE treatment in neuroendocrine cancer. This was assessed in a mouse NET xenograft model, in order to investigate effects on tumor growth, survival, and potential off-target effects from monotherapy and combination treatments. To our knowledge, this is the first study evaluating potential radiosensitizing effects of Onalespib with ^177^Lu-DOTATATE in an in vivo setting, as well as the potential effects on renal toxicity.

## Material and methods

### Cell lines

BON, established from a lymph node metastasis of a pancreatic carcinoid tumor, was kindly provided by Prof. Townsend (The University of Texas Medical Branch, Texas University, Galveston, TX, USA) and cultured in Dulbecco’s Modified Eagle Medium (DMEM)/Ham’s F12 1:1 (Biochrome, Germany). Squamous cell carcinoma cell line UM-SCC-74B was kindly provided by professor TE Carey (University of Michigan, USA) and cultured in DMEM. Cells were supplemented with 9% fetal bovine serum (Sigma Aldrich, St. Louis, USA), 1% l-glutamine, and 1% antibiotics (100 IU penicillin and 100 μg/mL streptomycin) from Biochrom (Germany). UM-SCC-74B was additionally supplemented with 1% non-essential amino acids (Biochrome, Germany).

### Drug and radioconjugate treatment

Onalespib (Selleckchem, Germany) was dissolved in DMSO and further diluted in 17.5% 2-hydroxypropyl beta-cyclodextrin (cyclodextrin) from Sigma-Aldrich (Germany) and injected intraperitoneally. For placebo injections, DMSO in 17.5% cyclodextrin was administered.

For labeling, DOTA-(Tyr^3^)-Octreotate (DOTATATE) (Bachem, Germany) was mixed with labeling buffer (25 mM sodium ascorbate/50 mM sodium acetate, pH 5). 40 MBq ^177^Lu (ITG, Germany) per microgram DOTATATE was added and subsequently incubated at 80 °C for 30 min. Labeling yield was assessed with instant thin layer chromatography (Biodex, New York, USA) with sodium citrate (0.1 M, pH 5.5) as mobile phase. A labeling yield of 99% or higher was used for all assays. ^177^Lu-DOTATATE was further diluted in 0.9% NaCl to 2 μg/mL and 50 μL was injected into the tail vein. For the tumor growth and survival study, injected activity per day was 4 MBq. For biodistribution assays, injected activity was 500 kBq and unlabeled DOTATATE was added to obtain 2 μg/mL. For imaging, the injected amount was 40 MBq with a concentration of 20 μg/mL. For placebo injections, 0.9% NaCl was administered.

### In vivo xenograft models

Female BALB/c nu/nu mice (*N* = 63, age = 4–6 weeks) were housed under standard laboratory conditions and fed ad libitum. 6 × 10^6^ BON cells in serum-free media were injected in the right flank. For biodistribution assay, 1 × 10^6^ UM-SCC-74B cells in serum-free media were injected in the left flank approximately 2 weeks later. Tumor diameter was measured using a digimatic caliper (Mitutoyo, Sweden) and volume was calculated as *4πabc/3* where *a*, *b*, and *c* were measured diameters in all dimensions. Mouse weight and tumor growth were monitored every other day.

### ^177^Lu-DOTATATE biodistribution

To verify antigen selectivity after labeling, biodistribution of ^177^Lu-DOTATATE was studied in mice bearing both BON (SSTR-positive) and UM-SCC-74B (SSTR-negative) xenografts (*N* = 4). Approximately 1 month after inoculation, 500 kBq ^177^Lu-DOTATATE (0.1 μg) was injected. Twenty-four hours post-injection, animals were sacrificed and organs were collected, weighed and radioactivity was measured in a gamma counter (Wallace, Finland).

### Ex vivo autoradiography

To investigate spatial distribution of ^177^Lu-DOTATATE after Onalespib treatment, autoradiography was performed on animals treated with either ^177^Lu-DOTATATE (*N* = 3) or the combination of Onalespib and ^177^Lu-DOTATATE (*N* = 3). The 4-day treatment regime consisted of a daily intra-peritoneal (i.p.) injection of 30 mg/kg Onalespib or placebo on days 1–4 and a daily intra-venous (i.v.) injection of 4 MBq ^177^Lu-DOTATATE (0.1 μg) on days 2–4. Onalespib and ^177^Lu-DOTATATE injections were given concomitantly. Forty-eight hours after last treatment, tumors were collected and embedded in O.C.T medium (VWR, Belgium). Tumors were subsequently sectioned with a microtome (20-μm sections) and the distribution of the remaining radioactivity was recorded with a phosphorimager (Fujifilm BAS-1800 II, Japan). ImageJ for Mac OSX version 1.48v (NIH, Bethesda, MD, USA) was used to quantify the distribution of activity in the tumor section [31]. Activity was defined as pixel intensity per tumor area in the phosporimager output file, on an arbitrary scale and normalized to control.

### In vivo tumor growth and survival

The effects of Onalespib, ^177^Lu-DOTATATE, or the combination of the two were studied in mice bearing BON tumors (*N* = 45). Upon visible tumors, measurement of tumor size by caliper was initiated and performed every 2 days throughout the study. At least two tumor measurements were performed prior to treatment start to verify established tumors. Personnel performing caliper measurements was blinded to the treatments. When tumors approached 50 mm^3^, animals were randomized into four groups: placebo (*N* = 15), Onalespib (*N* = 7), ^177^Lu-DOTATATE (*N* = 12), and combination (*N* = 7). Four animals were excluded from the study due to no visible tumor (*N* = 1) or too big tumor (*N* = 3) at treatment start. There were no significant differences in tumor starting volumes between the groups, with median sizes of 50, 30, 37, and 38 mm^3^ for control, Onalespib, ^177^Lu-DOTATATE, and combination groups respectively. The 4-day treatment regime consisted of a daily i.p. injection of 30 mg/kg Onalespib or placebo on days 1–4 and a daily i.v. injection of 4 MBq ^177^Lu-DOTATATE (0.1 μg) or placebo on days 2–4. Onalespib and ^177^Lu-DOTATATE injections were given concomitantly. The treatment regimen was selected through preceding dose escalation studies in BON xenografts (data not shown). Endpoint was set to a tumor size of 1 cm^3^ or weight loss of more than 10% compared with day of treatment start. Upon reaching endpoint, animals were sacrificed and the tumor, liver, and kidneys were collected and fixed in 4% buffered formalin for further analysis.

### Ex vivo immunohistochemistry

Ex vivo immunohistochemistry was performed to evaluate toxicity parameters and the molecular response to therapy. Mice bearing BON tumors were treated with placebo or with Onalespib and/or ^177^Lu-DOTATATE as previously described (*N* = 3 per group). Animals were sacrificed and organs were collected and fixed in 4% buffered formalin 48 h after last treatment. For study of toxicity 25 days after treatment, organs from the in vivo therapy study were collected as described above. Tissues were paraffin embedded, sectioned, and deparaffinized. Staining was performed with a Dako Autostainer 48 (Agilent, USA). Antigen retrieval was performed with retrieval solution low pH (Dako K8005, Agilent) or high pH (Dako K8004, Agilent). Sections were immunostained with antibodies against EGFR (1/200, Abcam, Sweden), VEGFR (1/400, Abcam), SSTR2 (1/1000, Abcam), or HSP70 (1/500, Sigma Aldrich) and detected with Envision Flex kit (Dako K8010, Agilent). Counterstaining with hematoxylin (Histolab, Sweden) was performed in a Tissue-Tek Prisma (Sakura, Netherlands). Immunohistochemical sections were manually scored according to staining intensity (negative −, weak +, moderate ++, or strong +++). For reference images, see figure S1. For xenografts, two or three random areas of 2 mm^2^ per section were scored and a median intensity as well as a median extent score was estimated. Extent score was defined on a scale of 0–3 (0 ≤ 25%, 25% < 1 ≤ 50%, 50% < 2 ≤ 75%, 3 > 75%). All histological analyses were performed in a blinded manner. Only tumor tissue was scored, stroma and necrotic areas were excluded from the analysis.

For toxicity analyses, four random areas of 2 mm^2^ on kidney and liver sections were chosen and presence/absence of damage was determined in a blinded manner. Furthermore, for kidneys, the percentage of damaged glomeruli, defined as significant glomeruli contraction, was quantified in each area in a blinded manner. Similarly, the presence/absence of HSP70 positive glomeruli and tubules was quantified in kidney sections taken 48 h after last treatment by counting positive or negatively stained structures within the four areas. Only tubules with a clearly defined lumen were included in the analysis. The staining intensity was quantified as described above.

### In vivo SPECT/CT imaging

SPECT/CT imaging was performed to confirm the complete remission of animals with no discernible tumor at study endpoint. For comparison, two untreated controls were also imaged when tumor reached 1 cm^3^. Animals were euthanized 24 h post-injection of 40 MBq of ^177^Lu-DOTATATE (1 μg) and imaged with a static whole-body tomographical scan in the NanoScan SPECT/CT (Mediso Medical Imaging Systems Ltd., Hungary). First, a whole-body CT scan was acquired with following parameters. Scan method: Semi circle FOV; projections 480; X-ray, 50 kVp and 600 μA; binning, 1:4; acquisition time, 2 min 47 s. SPECT scan was performed on same scan range as CT, for 60 min with following parameters. Frame time, 60 s; acquisition over 208.4, 112.90, and 56.10 keV. SPECT raw data were reconstructed in Nucline software (3.00.020.000) using TeraTomo 3D algorithm with 3 subsets, 48 iterations and corrected for scatter and attenuation artifacts. The CT raw files were reconstructed using Filter Back Projection. SPECT and CT dicom files were fused and analyzed using PMOD v3.510 (PMOD Technologies Ltd., Switzerland).

### Statistical analysis

Statistical analysis was performed with Graphpad Prism 7 for Mac (GraphPad Software, CA, USA) and *p* ≤ 0.05 was considered significant. Asterisks were used to specify significance level, where * denotes *p* ≤ 0.05, ** ≤ 0.01, *** ≤ 0.001, and **** ≤ 0.0001. For comparisons, one- or two-way ANOVA with Tukey’s or Dunnet’s multiple comparison was used. Growth curves were fitted to an exponential curve and start volume was set to 1. Kaplan-Meier survival curves were analyzed with Mantel-Cox test. For data in the text, mean and standard deviation is always presented unless otherwise stated.

## Results

### ^177^Lu-DOTATATE selectivity and distribution

**Fig. 1:**
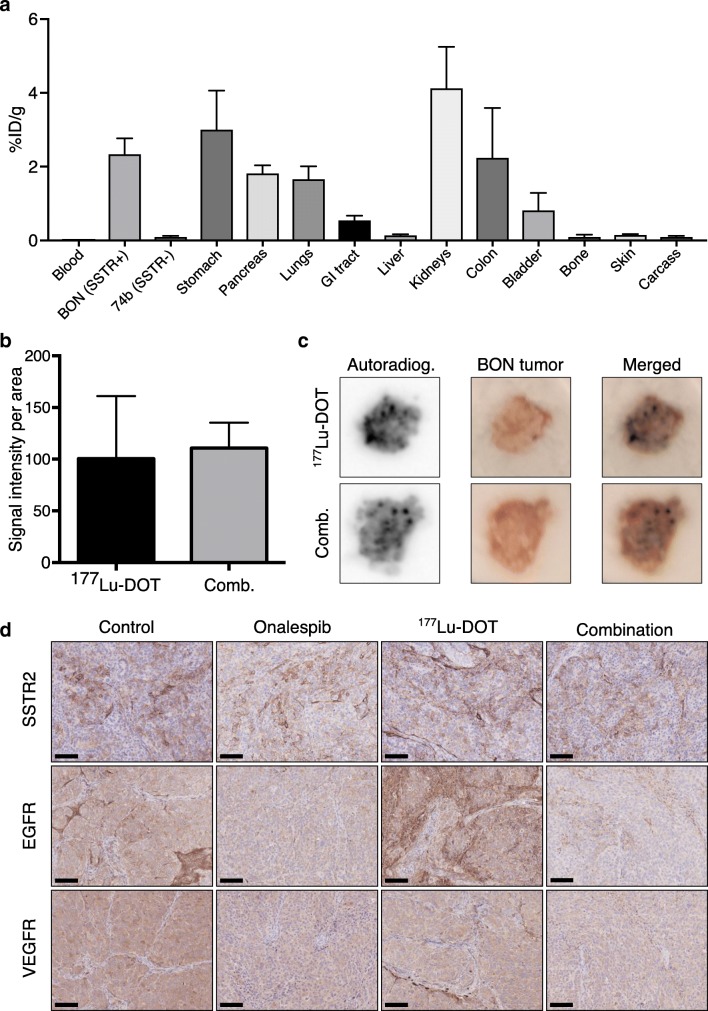
Ex vivo target validation. **a** Biodistribution of ^177^Lu-DOTATATE 24 h after injection of ^177^Lu-DOTATATE (mean, SD. *N* = 4). **b** Autoradiography quantification of BON tumor sections treated with either ^177^Lu-DOTATATE (black bar) or Onalespib combined with ^177^Lu-DOTATATE (gray bar). Graph displays mean, SD. *N* = 3. **c** Representative images from autoradiography (left), image of tumor (middle), and the merged image (right) of tumors treated with ^177^Lu-DOTATATE (top panel) or combination treatment (bottom panel). **d** Representative BON tumor images of immunohistochemical stainings of SSTR (top), EGFR (middle), and VEGR (bottom). Bar = 100 μm

To verify the antigen selectivity of ^177^Lu- DOTATATE, a biodistribution assay was performed 24 h post-injection. SSTR-expressing BON tumors exhibited 23 times higher uptake compared with SSTR-negative UM-SCC-74B tumors (Fig. 1a). High uptake was also found in kidneys, stomach, colon, pancreas, and lungs.

As Onalespib can influence angiogenesis and the expression of numerous client proteins [32, 33], the binding and spatial distribution of ^177^Lu-DOTATATE after Onalespib treatment as well as expression of SSTR2 was investigated. Ex vivo autoradiography of BON tumor sections 48 h after the last treatment revealed that Onalespib treatment did not alter the uptake of ^177^Lu-DOTATATE in the assessed sections (Fig. 1b). Moreover, spatial distribution was unchanged, with ^177^Lu-DOTATATE tumor uptake visible in the entire tumor sections, though highest in the vicinity of tumor blood vessels (Fig. 1c). Immunohistochemical stainings of SSTR2 were performed to further validate these results (Fig. 1d, Table 1). The staining intensity ranged from moderate to strong in all groups, with a median intensity of strong in control and combination groups and moderate in Onalespib and ^177^Lu-DOTATATE groups. All groups exhibited a median extent score of 1.

**Table 1:** Summary of immunohistochemical scoring of BON tumor sections with median intensity and extent scores for each treatment group. Immunohistochemical sections were scored according to staining intensity (negative −, weak +, moderate ++, or strong +++). For reference images, see figure S1. Extent score was defined on a scale of 0–3 (0 ≤ 25%, 25% < 1 ≤ 50%, 50% < 2 ≤ 75%, 3 > 75%)

Target	SSTR2		EGFR		VEGFR		HSP70	
	Intensity	Extent	Intensity	Extent	Intensity	Extent	Intensity	Extent
Control	+++	1	++	3	+++	3	++	3
Onalespib	++	1	+/++	3	+/++	3	++	3
^177^Lu-DOTATATE	++	1	+++	3	++/+++	3	+	2
Combination	+++	1	+/++	2	+	3	++	3

### Molecular response to treatment

EGFR and VEGFR are HSP90 client proteins and can be used to assess molecular responses after Onalespib therapy [15, 30, 33]. Here, the expression of EGFR and VEGFR was assessed in tumors with immunohistochemistry 48 h after the last treatment (Fig. 1d, Table 1). The untreated control group exhibited a moderate (++) EGFR staining intensity. Onalespib caused a slight decrease in staining intensity (+/++) while ^177^Lu-DOTATATE in contrast caused an increase (+++). The EGFR staining intensity in the combination group was similar to that of the Onalespib group (+/++). Extent scores ranged from 2 to 3 with a median score of 3 in control, Onalespib, and ^177^Lu-DOTATATE groups, while combination group demonstrated an extent score of 1–3 with median score of 2. VEGFR expression intensity was strong in the untreated control group (+++). The Onalespib group demonstrated weak to moderate VEGFR staining intensity (+/++). The levels in the ^177^Lu-DOTATATE group were slightly lower than those of the untreated control (++/+++), while the combination group demonstrated the lowest expression of VEGFR (+). All groups exhibited an extent score of 3 for VEGFR staining.

### In vivo therapy study

**Fig. 2:**
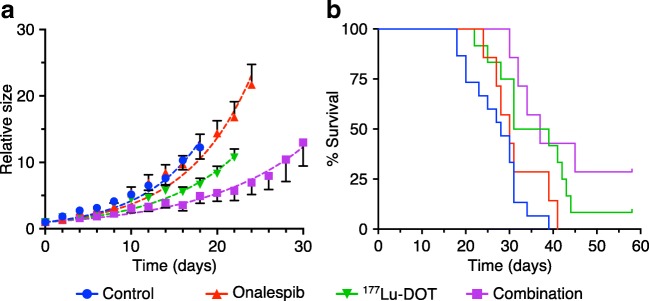
In vivo xenograft study. **a** Tumor growth over time (mean, SEM. *N* ≥ 7). Data was fitted to an exponential growth curve (dashed line). **b** Survival proportions of mice (*N* ≥ 7)

The efficacy of Onalespib- and ^177^Lu-DOTATATE monotherapy as well as the combination of the two was investigated in a xenograft model with neuroendocrine BON tumors (Fig. 2, fig. S2a). Onalespib significantly delayed tumor growth with a 10% delay in tumor doubling time from 4.8 days in control group to 5.3 days (Table 2). ^177^Lu-DOTATATE and combination treatment resulted in further prolongation of tumor doubling time to 6.4 and 8.3 days, corresponding to a delay of 33% and 73%, respectively. The increased growth delay correlated with an increase in survival (Fig. 2b, Table 2) with Onalespib monotherapy slightly affecting median survival (30 days compared with 28 days in control group). ^177^Lu-DOTATATE and combination groups demonstrated greater effects, with an increased survival to 35 and 37 days, respectively.

**Table 2:** Summary of in vivo tumor growth and survival study. Tumor doubling time (95% CI) was calculated from the curve fit obtained from data in Fig. 2a

Treatment	Control	Onalespib	^177^Lu-DOT	Combination
Tumor doubling time (days)	4.8 (4.7–5.0)	5.3 (5.2–5.5)	6.4 (6.2–6.6)	8.3 (7.9–8.8)
Median survival (days)	28	30	35	37
Complete response (%)	0	0	8	29

**Fig. 3:**
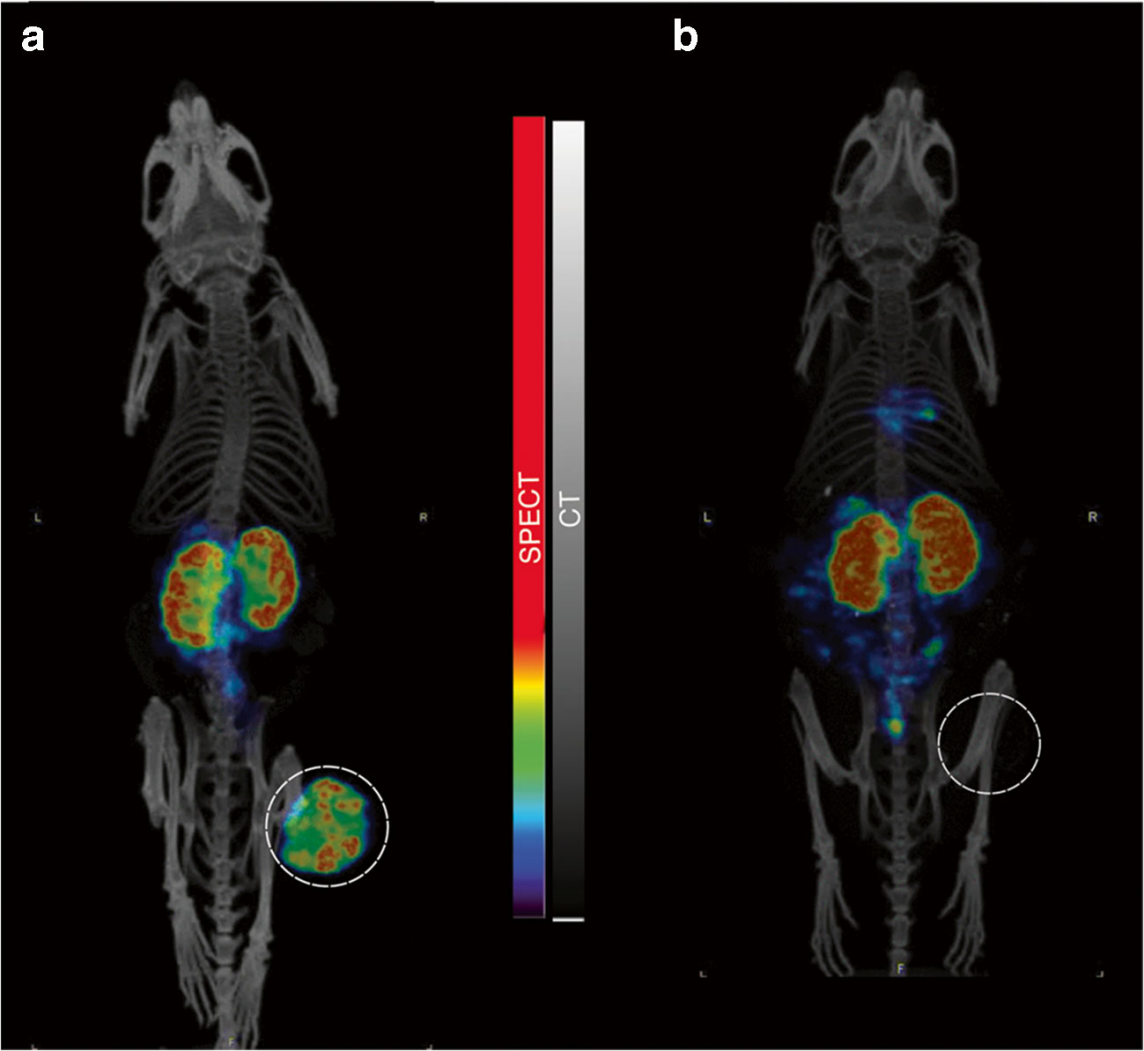
Representative SPECT/CT images at endpoint for mouse sacrificed due to size of tumor (**a**) and mouse reaching complete remission (**b**)

No animals in the control or Onalespib groups demonstrated complete remissions. However, the ^177^Lu-DOTATATE and combination therapy groups were able to completely eradicate the tumors in several animals, resulting in 8% complete remissions in the ^177^Lu-DOTATATE group and 29% complete remissions in the combination group. SPECT/CT imaging with ^177^Lu-DOTATATE (Fig. 3) and dissection confirmed no residual tumor tissue present in these mice.

There was no correlation between tumor volume at treatment start and complete remissions, where tumor volumes at treatment start for animals with complete remissions stretched between the 10th and 60th percentile. Moreover, the treatments did not affect mouse weight negatively (fig. S2b).

### Toxicity profile

**Fig. 4:**
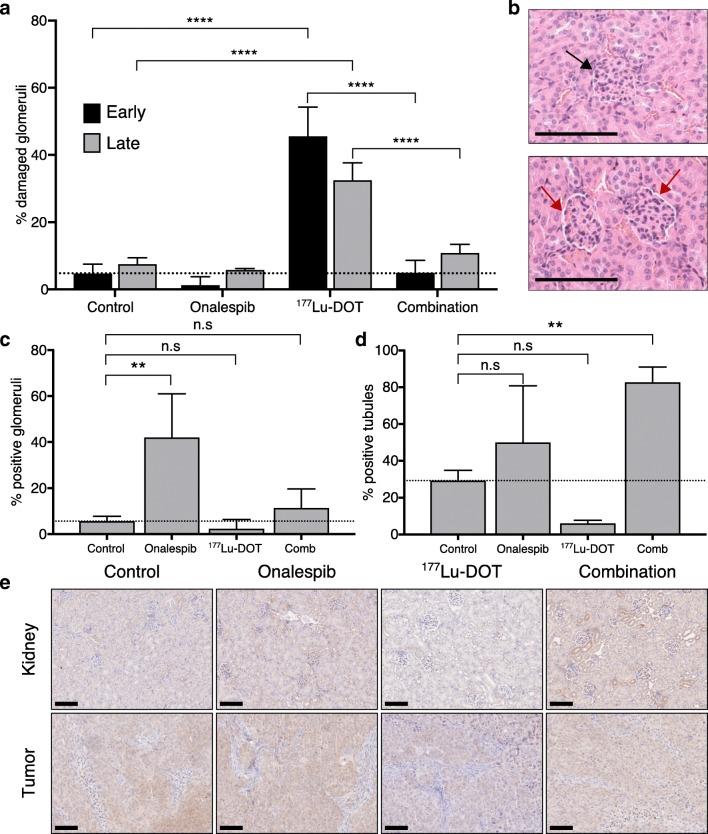
Ex vivo immunohistochemical and histological analysis. **a** Quantification of glomerular damage (mean, SD. *N* = 3). **b** Representative images of a normal (top image, black arrow) and damaged (bottom image, red arrows) glomeruli. Bar = 100 μm. **c** Quantification of staining extent of HSP70 positive glomeruli (mean, SD. *N* = 3). **d** Quantification of staining extent of HSP70 positive tubules (mean, SD. *N* = 3). **e** Representative images of immunohistochemical stainings of HSP70 in kidneys (top) and tumors (bottom). Bar = 100 μm

In order to define a liver and kidney toxicity profile of Onalespib, ^177^Lu-DOTATATE, and the combination of the two, the liver and kidneys were collected 48 h and 25 days post-treatment. Evaluation of hematoxylin and eosin staining of liver tissue revealed no signs of hepatotoxicity in any of the treatment groups (data not shown). Likewise, no signs of tubular damage in the kidneys were found in any of the treatment groups (data not shown). When considering the glomerular contraction (Fig. 4a, b), the untreated controls demonstrated a similar percentage of affected glomeruli at 48 h (5% ± 3) and 25 days (8% ± 2) post-treatment. Onalespib did not induce a significant change in glomeruli damage compared with untreated controls. ^177^Lu-DOTATATE, however, caused a 10-fold increase in the number of damaged glomeruli (46% ± 9) on day 2 compared with untreated controls. The extent of the damage was slightly lower on day 25 (33% ± 5), but still significantly higher than control group. In the combination group, the damage was absent and the levels of glomeruli contraction, 5% ± 4 at 48 h and 11% ± 2 at 25 days, were comparable with those of the untreated control and Onalespib groups.

Immunohistochemical analyses of HSP70 kidney expression 48 h after treatment in glomeruli and tubules were performed to investigate HSP70 involvement (Fig. 4c–e). For glomeruli, the median staining intensity was slightly higher in Onalespib- and combination-treated groups (+) compared with untreated control and ^177^Lu-DOTATATE (−/+), but the extent differed more (Fig. 4c). Untreated control and ^177^Lu-DOTATATE groups demonstrated low levels of HSP70 positive glomeruli, 6% (± 2) and 2% (± 4), respectively. A significant increase in staining extent was seen in the Onalespib group, with 42% (± 19) positive glomeruli. The combination-treated group followed the same trend with 11% (± 8) positive glomeruli, however not statistically significant.

For tubules (Fig. 4d), the extent of HSP70 expression followed a similar pattern as for glomeruli. In untreated controls, the extent of HSP70 positive tubules was 29% (± 6). For ^177^Lu-DOTATATE, only 6% (± 2) of the tubules were HSP70 positive, although this reduction was not statistically significant compared with controls. In the Onalespib group, the extent varied between animals, with a mean value of 50% (± 31), whereas in the combination group, a significant increase to 83% (± 8) was observed.

To relate the expression patterns in kidneys to those in tumors, xenografts were also stained for HSP70 (Fig. 4e, Table 1). The expression was heterogeneous within groups but also within separate tumor sections. The intensities ranged from negative to strong with median staining intensity of moderate (++) in all groups, except for the ^177^Lu-DOTATATE group, which demonstrated a weak staining intensity (+). The median extent scores for untreated control, Onalespib and ^177^Lu-DOTATATE groups were 3 while combination group had a score of 2.

## Discussion

^177^Lu-DOTATATE has revolutionized the treatment of inoperable NETs, but complete response is still rare [3]. Radiosensitizing drugs that potentiate the effects of radiotherapy have the potential to increase treatment efficacy without causing undesirable toxicity [14]. We have previously demonstrated that there is great potential in combining Onalespib with ^177^Lu-DOTATATE in vitro [30]. Consequently, we hypothesized that HSP90 inhibitor Onalespib could potentiate ^177^Lu-DOTATATE treatment of neuroendocrine tumors in vivo.

The antigen selectivity of ^177^Lu-DOTATATE was first evaluated in a dual xenograft model, where uptake in an SSTR-expressing tumor was compared with a negative control tumor (Fig. 1a). Results demonstrated an uptake in SSTR-positive tumors 23 times higher than in SSTR-negative tumors. The results of the biodistribution were in line with the results of previous studies [34–37]. Autoradiography (Fig. 1b, c) and immunohistochemistry (Fig. 1d, Table 1) were performed to determine whether Onalespib treatment altered SSTR2 expression, thereby potentially affecting the uptake and distribution of ^177^Lu-DOTATATE. No difference between the groups was observed by autoradiography, and only slight differences in SSTR2 staining intensity were observed with immunohistochemistry, but this did not correlate with Onalespib treatment. Overall, these results confirm our previous in vitro results demonstrating that SSTR is not a client protein of HSP90 [30]. Consequently, these characterizations validated the in vivo selectivity of ^177^Lu-DOTATATE, the relevance of the animal model used, and the in vivo feasibility of combining Onalespib with ^177^Lu-DOTATATE.

To characterize molecular effects in vivo from the treatment, immunohistochemistry was performed on tumors collected 48 h after treatment. EGFR and VEGFR are both client proteins of HSP90 and can be used as markers for Onalespib response [17]. Downregulation of both markers was found in Onalespib- and combination-treated tumors (Fig. 1d, Table 1) confirming a molecular response to the Onalespib treatment. These results are consistent with previous in vitro studies, demonstrating Onalespib-induced downregulation of EFGR and VEGFR [15, 30]. Furthermore, ^177^Lu-DOTATATE caused a slight upregulation of EGFR. A finding that needs to be further verified, but if proven accurate, it may explain some of the mechanisms behind the success of the combination therapy, as increased EGFR signaling can result in sustained proliferation [38]. Since Onalespib has the ability to suppress the EGFR upregulation, this may be one of several factors contributing to the better treatment response in the combination group.

In the in vivo therapy study, Onalespib and ^177^Lu-DOTATATE demonstrated significant effects on tumor growth as monotherapies, delaying tumor doubling time from 4.8 days in untreated controls to 5.3 and 6.4 days respectively (Fig. 2). However, the most pronounced effects were seen in the combination group, where tumor doubling time was delayed to 8.3 days. These results are in line with a recent study, where the HSP90 inhibitor Ganetespib demonstrated radiosensitizing effects in small intestinal neuroendocrine tumors [16].

The delayed tumor doubling times were also in accordance with the survival data (Fig. 2b, Table 2), where the combination group displayed the greatest effect with a prolonged median survival of more than 30% from controls. Moreover, complete remissions increased to 29%, compared with 8% in the ^177^Lu-DOTATATE group and 0% in the Onalespib group (Fig. 2b). Complete remissions were further validated at the endpoint of the study through SPECT/CT analysis (Fig. 3) and dissections. Thus, we deduce that Onalespib is able to potentiate ^177^Lu-DOTATATE, demonstrated by delayed tumor growth, increased median survival, and most notably by a threefold increase in complete remissions from ^177^Lu-DOTATATE monotherapy. These results are encouraging and indicate that the addition of Onalespib may aid in increasing curative rates from PRRT. Additional optimizations in Onalespib dosage, fractionations, and timing with ^177^Lu-DOTATATE treatment should be explored in order to further improve results.

Toxicity is an important issue when combining therapies. In the case of PRRT, renal toxicity is a dose-limiting factor that must be considered. In the current clinical treatment regime of NET patients treated with ^177^Lu-DOTATATE, co-infusion with positively charged amino acids is used to lower tubular reabsorption of the radiopeptide. However, amino acid co-infusion does not protect from damages to the glomeruli, through which ^177^Lu-DOTATATE must be filtrated in order to be cleared [13]. In the present study, we demonstrate a clear reduction in glomeruli contraction in combination-treated animals compared with animals given only ^177^Lu-DOTATATE (Fig. 4b). Although the damage was not severe, a clear distinction between damaged and undamaged glomeruli could be observed (Fig. 4c). In comparison, studies conducted with higher doses of ^177^Lu-DOTATATE renal toxicity in rodents have exhibited similar patterns, although with a more severe toxicity profile [39]. Intriguingly, these results indicate that while Onalespib induces a radiosensitizing effect in the tumors, a radioprotective effect may be generated in the kidneys. We hypothesize that HSP90 maintains a more prominent role in the tumor than in normal tissue, and thus inhibition of this protein significantly reduces survival to a larger extent in the tumor cells. In contrast, HSP70, which can be upregulated as a response to HSP90 inhibition [19–21], has been shown to play a prominent role in kidneys and may provide an explanation for the renal protective effects.

Consequently, to further unravel the causes of the observed protective effects in kidneys, immunohistochemical analysis was performed to quantify the HSP70 expression after the different treatments (Fig. 4c–e). The results verified increased renal HSP70 expression in both Onalespib- and combination-treated groups. These results are in line with previous studies demonstrating that HSP90 inhibition can induce HSP70 expression [19–21], and agree with studies revealing that HSP70 constitutes a component of the endogenous stress response to renal injury [22–24]. HSP70 can be induced in both glomeruli and proximal tubules, and has demonstrated a cytoprotective role in kidneys, e.g., through chaperoning actions or antioxidative properties. Interestingly, HSP70 expression in tumors assessed at the same time point did not differ much between the treatment groups (Fig. 4e), with the exception of the ^177^Lu-DOTATATE group, which demonstrated lower levels of HSP70. It is possible that the kinetic profiles of HSP70 inductions differ between kidneys and tumors. Thus, we deduce that Onalespib-induced upregulation of HSP70 in kidneys may act as a radioprotector, although more detailed kinetic studies on HSP70 expression in both kidneys and tumors would be recommended to further investigate these findings. Further, to what extent Onalespib treatment affects the bone marrow, which is a dose-limiting organ in ^177^Lu-DOTATATE therapy, has not been addressed in this study. However, no bone marrow toxicity was reported in the clinical studies conducted with Onalespib.

In conclusion, we demonstrate that the HSP90 inhibitor Onalespib potentiates ^177^Lu-DOTATATE in a neuroendocrine tumor xenograft model, resulting in delayed tumor growth, increased complete remissions, and reduced renal toxicity. Our findings demonstrate the feasibility of Onalespib as a therapeutic option and radiosensitizer for neuroendocrine tumors, as well as a potential kidney protective agent. If further validated, the combination of Onalespib and ^177^Lu-DOTATATE may in the future lead to increased cure rates in PRRT while improving the toxicity profile.

## Electronic supplementary material

Fig. S1 Reference images of staining intensities for immunohistochemical analysis. Bar =50 μm (PNG 536 kb)

High Resolution Image (EPS 12460 kb)

Fig. S2 Longitudinal presentation of A) relative tumor growth over time of each individual mouse B) Relative mouse weight over time (mean, range. N ≥ 7) (PNG 167 kb)

High Resolution Image (EPS 81 kb)
