# Supplementary figures and images for: The radiosensitizer Onalespib increases complete remission in 177Lu-DOTATATE-treated mice bearing neuroendocrine tumor xenografts
Source: Eur J Nucl Med Mol Imaging. 2020 Jan 7;47(4):980–90. doi: 10.1007/s00259-019-04673-1 (PMC7075859; doi:10.1007/s00259-019-04673-1)

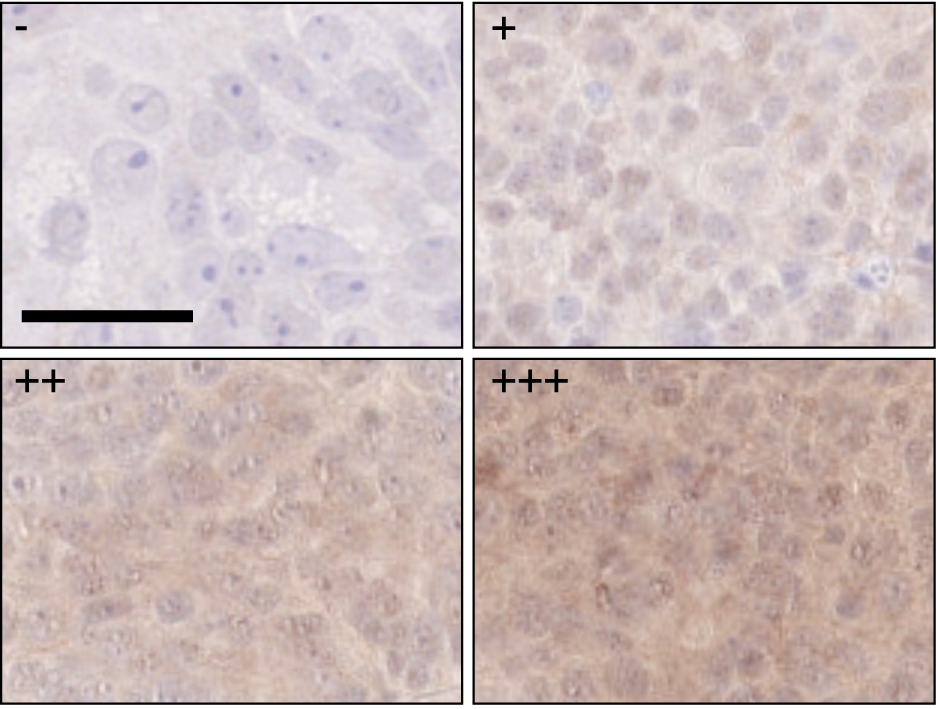

Supplement: Supplementary file 1 — Reference images of staining intensities for immunohistochemical analysis. Bar =50 μm (PNG 536 kb) [file 259_2019_4673_Fig5_ESM.png]

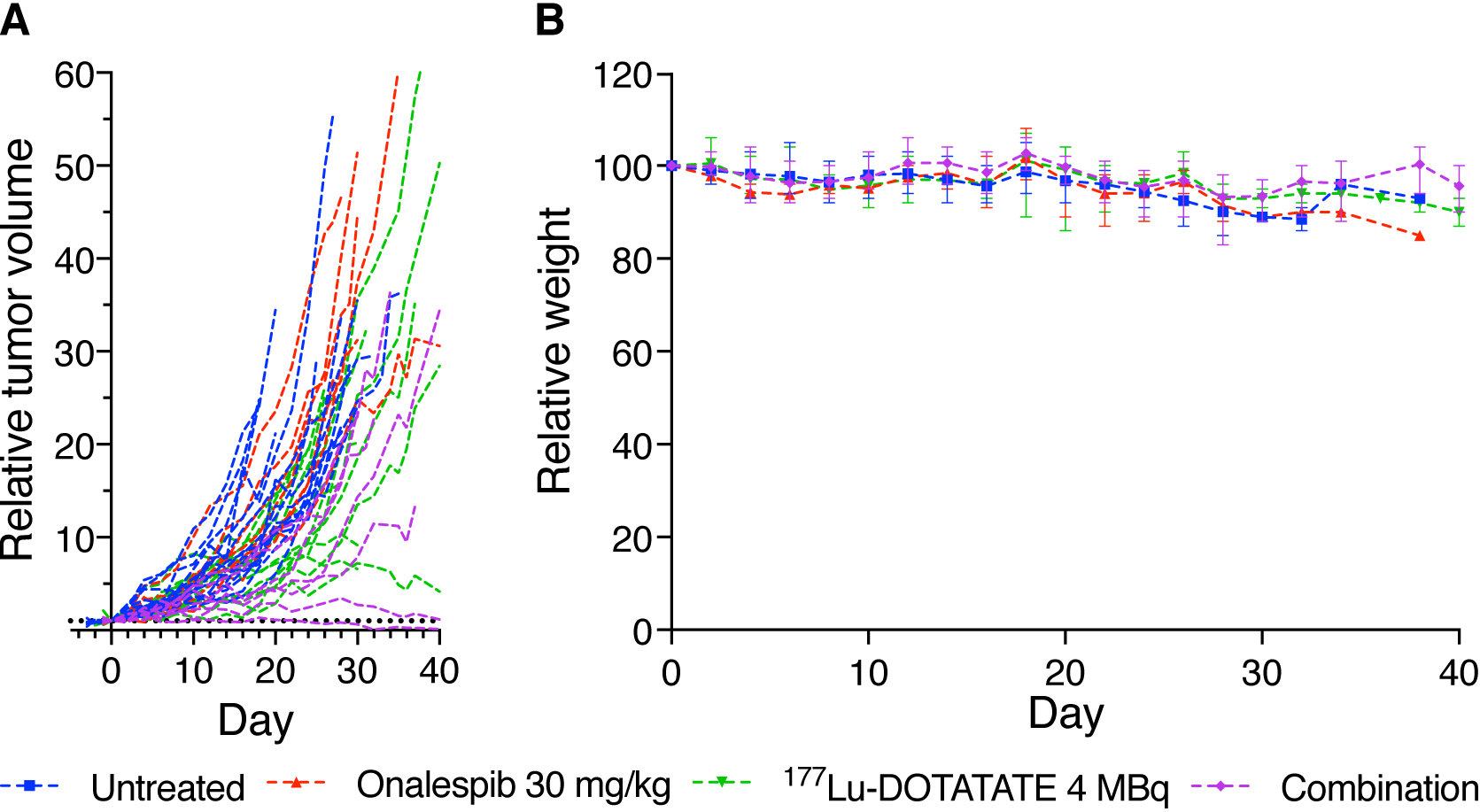

Supplement: Supplementary file 3 — Longitudinal presentation of A) relative tumor growth over time of each individual mouse B) Relative mouse weight over time (mean, range. N ≥ 7) (PNG 167 kb) [file 259_2019_4673_Fig6_ESM.png]
